# Does DDI-Predictor Help Pharmacists to Detect Drug-Drug Interactions and Resolve Medication Issues More Effectively?

**DOI:** 10.3390/metabo11030173

**Published:** 2021-03-17

**Authors:** Fanny Moreau, Nicolas Simon, Julia Walther, Mathilde Dambrine, Gaetan Kosmalski, Stéphanie Genay, Maxime Perez, Dominique Lecoutre, Stéphanie Belaiche, Chloé Rousselière, Michel Tod, Bertrand Décaudin, Pascal Odou

**Affiliations:** 1 Institut de Pharmacie, CHU Lille, F-59000 Lille, France; fanny.moreau@chru-lille.fr (F.M.); julia.walther@outlook.fr (J.W.); mathilde.dambrine@chru-lille.fr (M.D.); gkosmalski@ch-luneville.fr (G.K.); stephanie.genay@chru-lille.fr (S.G.); maxime.perez@chru-lille.fr (M.P.); dominique.lecoutre@chru-lille.fr (D.L.); stephanie.belaiche@chru-lille.fr (S.B.); chloe.rousseliere@chru-lille.fr (C.R.); bertrand.decaudin@univ-lille.fr (B.D.); pascal.odou@univ-lille.fr (P.O.); 2 ULR 7365–GRITA–Groupe de Recherche sur les formes Injectables et les Technologies Associées, University of Lille, F-59000 Lille, France; 3 EMR: 3738, Faculté de Médecin Lyon-Sud-Charles Mérieux, Université Lyon 1, F-69921 Oullins, France; michel.tod@univ-lyon1.fr; 4 Pharmacie, Groupement Hospitalier Nord, Hospices Civils de Lyon, F-69005 Lyon, France

**Keywords:** medication analysis, drug-drug interaction, pharmaceutical care, prevention

## Abstract

The characterization of drug-drug interactions (DDIs) may require the use of several different tools, such as the thesaurus issued by our national health agency (i.e., ANSM), the metabolic pathways table from the Geneva University Hospital (GUH), and DDI-Predictor (DDI-P). We sought to (i) compare the three tools’ respective abilities to detect DDIs in routine clinical practice and (ii) measure the pharmacist intervention rate (PIR) and physician acceptance rate (PAR) associated with the use of DDI-P. The three tools’ respective DDI detection rates (in %) were measured. The PIRs and PARs were compared by using the area under the curve ratio given by DDI-P (R_AUC_) and applying a chi-squared test. The DDI detection rates differed significantly: 40.0%, 76.5%, and 85.2% for ANSM (The National Agency for the Safety of Medicines and Health Products), GUH and DDI-P, respectively (*p* < 0.0001). The PIR differed significantly according to the DDI-P’s R_AUC_: 90.0%, 44.2% and 75.0% for R_AUC_ ≤ 0.5; R_AUC_ 0.5–2 and R_AUC_ > 2, respectively (*p* < 0.001). The overall PAR was 85.1% and did not appear to depend on the R_AUC_ category (*p* = 0.729). Our results showed that more pharmacist interventions were issued when details of the strength of the DDI were available. The three tools can be used in a complementary manner, with a view to refining medication adjustments.

## 1. Introduction

Drug error is a major concern for inpatients and outpatients. It has been reported that serious adverse drug reactions were responsible for 6.5% of all admissions to two large general hospitals in the UK [1]. Similarly, two French studies [2,3] showed that drugs were responsible for almost 0.7‰ of serious adverse events and accounted for 4.5% of all hospital admissions [2,3].

To reduce the risk of adverse drug reactions, prescribing physicians must take account of several variables: compliance with the indication, the regimen, possible comorbidities (e.g., renal, hepatic or cardiac failure, individual metabolic particularity) associated with pharmacokinetic changes, and potential drug-drug interactions (DDIs) [4].

Pharmacokinetic changes are difficult to quantify but can occur at different stages in the ADME process. Most (but not all) biotransformations are performed by cytochromes P450 (CYP). The CYP3A4, 2D6, 2C9, 1A2, 2C19 and 2E1 isoforms are responsible for metabolizing 80% of all drugs [5]. Given that the CYPs are genetically regulated protein complexes, their activity can be induced or inhibited by various compounds and various liver disorders (e.g., cirrhosis). Moreover, gene polymorphisms in certain isoforms (notably CYP2D6, CYP2C19, and CYP2C9) are potentially responsible for iatrogenic disorders (i.e., abnormally high or low metabolic activity) in treated patients [6]. These modifications may result in under- or over-dosing, which in turn can reduce drug effectiveness or lead to adverse events. It has been reported that between 10% and 30% of DDIs lead to adverse events [2,3,7] and that DDIs might account for up to 10% of emergency room admissions [8]. Pharmacogenetic modifications or cirrhosis might also lead to (sometimes serious) adverse events [9,10,11].

Pharmacists can use several tools to detect and evaluate DDIs [12]. Various DDI screening programs are commercially available and can be used to decide on dose adjustments or medication changes [12]. The programs’ sensitivity for detecting clinically relevant DDIs has been reviewed [12]. Tools that screen for DDIs must also help physicians or pharmacists to give accurate advice and thus optimize a patient’s medications. Some of these DDI checkers have been evaluated in the literature [13,14]. In France, clinical pharmacists usually look at a drug’s summary of product characteristics (SPC), together with regulatory guidelines issued by French national health agency (*Agence Nationale de Sécurité du Médicament et des Produits de Santé*, ANSM, Saint-Denis, France) [15]. A number of other tools can provide more details, such as the tables summarizing DDIs and the main metabolic pathways published by Geneva University Hospital (GUH, Geneva, Switzerland) [16].

DDI-Predictor (www.ddi-predictor.org, accessed on 18 January 2021) is a recently developed, free, online decision-making tool for characterizing pharmacokinetic modifications that involve the main CYPs; it notably takes account of possible cirrhosis or gene polymorphisms in the patient [17]. Using a mathematical model based on the DDIs observed in humans, DDI-Predictor’s output is quantified as the ratio of the area under the drug concentration curve (AUC), relative to that of a “standard” patient (R_AUC_) [18,19]. The AUC corresponds to the change over time in the drug’s concentration in the body. Hence, R_AUC_ will be greater than 1 in patients with enzyme inhibition, cirrhosis, or a gene polymorphism that slows drug metabolism. In contrast, R_AUC_ will be less than 1 in cases of enzyme induction or a gene polymorphism that accelerates drug metabolism. The opposite is true for pro-drugs. The size of the effect on R_AUC_ gives the pharmacist an idea of the importance of the phenomenon and helps him/her to advise the physicians on possible medication adjustments.

The goal of the pharmacist’s analysis is to reduce the occurrence of drug-associated iatrogenic events [20]. However, a lack of data on certain situations (such as DDIs, cirrhosis, or the presence of gene polymorphism) may prevent pharmacist from advising the physician on medication adjustments. National and international standards, specific online applications, and SPCs can help the pharmacist to detect potential DDIs or potential changes in drug metabolism associated with cirrhosis or gene mutations. Although DDI screening tools may classify the level of risk (e.g., contra-indication, an at-risk combination, or administration with caution), they rarely indicate how the doses should be modified. For the last few years, we have been using the GUH tools and DDI-Predictor in our routine practice, along with the ANSM thesaurus.

The primary objective of the present study was to compare the three reference tools’ abilities to detect DDIs. The secondary objective was to measure the pharmacist intervention rate (PIR) and the physician acceptance rate (PAR) following the use of DDI-Predictor.

## 2. Results

### 2.1. Description of the Interactions

**Figure 1:**
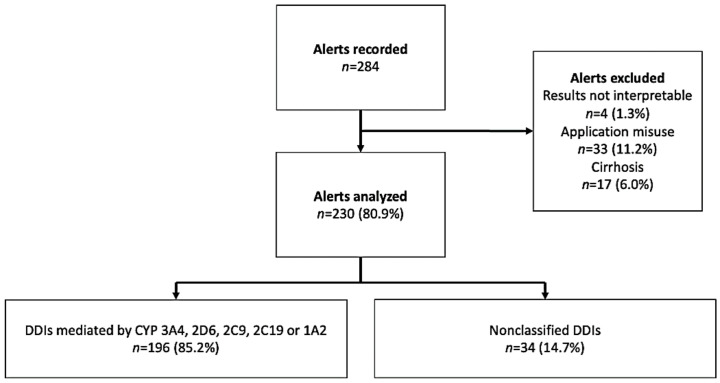
Study flow chart for DDIs.

Over a 24-month period, a total of 199,000 drug prescriptions were analyzed, and 284 alerts involving DDI-Predictor (i.e., potential DDIs) were generated (Figure 1). 88 alerts were excluded because of application misuse (*n* = 33), noninterpretable results (*n* = 4), or the presence of cirrhosis (*n* = 17). A total of 230 alerts were included in the final analysis.

The tools’ detection rates differed significantly: 92 out of 230 (40.0%), 176 out of 230 (76.5%), and 196 out of 230 (85.2%) for the ANSM thesaurus, the GUH table, and DDI-Predictor, respectively (*p* < 0.0001). The results are summarized for inducers and inhibitors in Table 1, with the ANSM thesaurus as the reference.

All four types of DDI defined in the ANSM thesaurus (taken as the reference) were detected by DDI-Predictor, including the four contraindicated drug combinations. Only two of the four were detected by the GUH table. Of the 22 at-risk drug combinations, 20 were detected by the GUH table and 19 were detected by DDI-P. Of the 44 drug combinations to be administered with caution, 27 were detected by the GUH table and 36 were detected by DDI-P. Of the 22 interactions to be taken into consideration, 19 were detected by the GUH table and 20 were detected by DDI-P. Lastly, 138 DDIs were not detected or not recorded by the ANSM thesaurus; of these, 99 were detected by the GUH table and 117 were detected by DDI-P.

### 2.2. Description of the PIs and PAR Associated with DDI-Predictor

After removing 34 alerts that could not be confirmed as DDIs, we analyzed 196 alerts (Figure 1).

Overall, the 196 alerts triggered 121 PIs (i.e., PIR = 61.7%). The PIR differed as a function of R_AUC_: 90.0%, 44.2% and 75.0% for R_AUC_ ≤ 0.5; R_AUC_ 0.5–2 and R_AUC_ > 2, respectively (*p* < 0.001). The overall PAR was 85.1% (103 out of the 121 PIs) (Table 2). The intergroup differences in the PAR were not significant (*p* = 0.729). The PIR for at-risk DDIs (R_AUC_ ≤ 0.5 and R_AUC_ > 2) was 81.5% (75 out of 92), and the PAR in this subgroup of PIs was 85.3% (64 out of 75).

A retrospective analysis of the PI and PAR associated with DDI-Predictor highlighted differences with regard to two other tools (Table 3).

Regardless of the R_AUC_ group, the PIR was higher for the GUH table than for the ANSM thesaurus. Overall, the PIR was 81.0% for the GUH table and 41.3% for the ANSM thesaurus (*p* < 0.0001).

The PIs issued with DDI-Predictor but not detected by the two other tools depended on the R_AUC_. For R_AUC_ ≤ 0.5, 43% of the DDI-Predictor’s PIs would have been issued with the ANSM thesaurus (drug changes because of interactions with rifampicin or carbamazepine); the PAR was 82%. 55% of the DDI-Predictor’s PIs would have been issued by the GUH (drug changes because of interactions with rifampicin or dabrafenib); the PAR was 100%. For R_AUC_ > 2, 44% of the DDI-Predictor’s PIs would have been issued by the ANSM thesaurus (dose decreases for drug combinations with fluoxetine and paroxetine); the PAR was 87%. Use of the GUH table led to PIs for fluoxetine, paroxetine, and grapefruit juice; the PAR was 66%.

Lastly, an analysis of alerts with R_AUC_ 0.5–2 revealed that the interactors were mainly strong inhibitors (i.e., fluconazole, paroxetine, fluoxetine, clarithromycin, or amlodipine) or strong inducers (i.e., rifampicin). The majority of these PIs advised the physician to monitor for adverse drug reactions because of the risk of overdosing. The PAR for this subset of PIs was over 80%.

## 3. Discussion

Medication analyses by a pharmacist help to prevent adverse drug reactions. However, the data given to pharmacists by several databases are rarely accurate enough to allow drug therapy to be safely modified in cases of a DDI, cirrhosis, or the presence of gene polymorphism.

Drug-drug interactions with a true clinical impact are not frequent [21] but require pharmacists to be vigilant. [22] In the present study, 92 of the 196 of the DDI-Predictor’s alerts (i.e., more than 40%) were considered to be significant (R_AUC_ ≤ 0.5 or >2) by the clinical pharmacist. More than 80% of the alerts results in a PI. Cases without a PI concern situations with limited clinical impact, a slight change in the AUC, the presence of several interacting drugs in the same prescription, or another cause of pharmacokinetic alterations (e.g., obesity or kidney failure). These complex cases will prompt the pharmacist and the physician monitor the patient closely regarding to the difficulty to advise any PI.

In our department, the pharmacist first analyses DDIs by reference to the ANSM thesaurus. The GUH table was published (and thus implemented) before DDI-Predictor. Our results show that the three tools are complementary–partly because they have different objectives. The ANSM thesaurus lists DDIs considered to be clinically significant [15]. The GUH table summarizes in vivo and in vitro data on potential biotransformation pathways or drug interactions [16]. Rapidly analyzable data improve the pharmacist’s analysis. DDI-Predictor’s output (R_AUC_) is calculated using physiologically based pharmacokinetic based models, which gives the pharmacist and idea of the “strength” of a DDI. Our results showed that pharmacists were more inclined to issue a PI when details of the DDI were available. It has been previously shown that the implementation of tools that help pharmacists to detect and prevent DDIs improves the management of drug therapy-notably in elderly patients [13]; our present results confirmed this. Moreover, the PIR and PAR values in Beeler et al.’s study of an on-demand DDI checker were similar to those observed in our study [14].

Indeed, a significant R_AUC_ prompted the pharmacist to issue a PI more readily and was associated with a high PAR. Both the ANSM thesaurus and the GUH table can detect DDIs with a limited clinical impact (R_AUC_ 0.5–2). In most cases considered to be at-risk combinations by the ANSM thesaurus, the PI recommended monitoring for side effects or therapeutic drug monitoring. Our results appear to show that few PIs in this group had a significant influence on patient care. Therefore, knowledge of the value of RAUC probably helped the pharmacist to best advise the physician on treatment changes.

Although DDI-Predictor detects contraindicated combinations listed in the ANSM thesaurus, the detection of other “strong” DDIs differed significantly. Hence, DDI screening tools should be combined in order to better detect at-risk drug combinations and thus prevent adverse drug reactions. Indeed, more than the half of the PIs based on DDI-Predictor were not detected by the ANSM thesaurus. The PAR in this subgroup of events was over 80%. Our results emphasize the tools’ complementarity with regard to their different objectives and the regularity of their updates, as already pointed out in a literature review [12]. Clinical decision support systems alert pharmacists and physicians to adverse drug reactions and medication errors [23]. Pharmacists can use these tools to define rules for the detection of pharmacokinetic changes (including DDIs), issue PIs, and thus improve patient safety [24]. Additional data from DDI-Predictor could be usefully included in this process.

Nonetheless, the use of DDI-Predictor has several limitations. Firstly, its use depends on the pharmacist’s level of knowledge of the interacting agents. Most of the recorded interactions involved very frequently prescribed drugs, and many are also well-known interactors: carbamazepine [25], rifampicin [26], paroxetine [27,28], fluoxetine [27], and fluconazole [29]. It is crucial to monitor co-prescribed drugs when judging the significance of interactions. The fact that the pharmacists were very aware of these drugs’ interactor potential might explain (at least in part) their high frequency in the database, relative to drugs with lesser known interacting effects. Indeed, some DDIs might have been omitted due to a lack of awareness, especially if the drugs are now frequently prescribed. This is why reviewing PIs can also help raise awareness of DDIs among pharmacists. Although the lists of drugs change continuously, the application is limited in the testable cases. Thus, DDI-Predictor cannot analyze all DDIs.

A high proportion of the initially detected alerts (42 out of 284 (14.7%)) could not be classified. This might be due to (i) the lack of published data in humans or (ii) drug metabolism by enzymes other than CYPs (e.g., the production of glucuronated or sulfonated metabolites or substrates for P-glycoprotein or organic anion transporters (e.g., OATP2B1)) [28]. Further development of DDI-Predictor might resolve these issues. DDI-Predictor’s algorithm computes R_AUC_ on the basis of linear kinetics, which is a poor approximation for drugs like phenytoin and voriconazole [29]. DDI-Predictor might complement other databases and thus improve DDI screening.

In the present study, DDI-Predictor was applied to a small proportion of the analyzed prescriptions. New tools have a learning curve. Some data may not be recorded, and some interactions may not be detected by pharmacists; this is also a study limitation. The underuse of DDI-Predictor can be also explained by the fact that some detected DDIs involved well tolerated home medications. In the absence of poor efficacy or toxicity, PIs were not issued.

Lastly, some misuses of DDI-Predictor were identified. Indeed, using the wrong R_AUC_ value or a R_AUC_ computed under different condition might lead to incorrect advice. Pharmacists must therefore be well trained in use of this application and must receive well-structured quality assurance documents. Initial and continuing education and training are essential.

## 4. Materials and Methods

### 4.1. The DDI Process Screening

**Figure 2:**
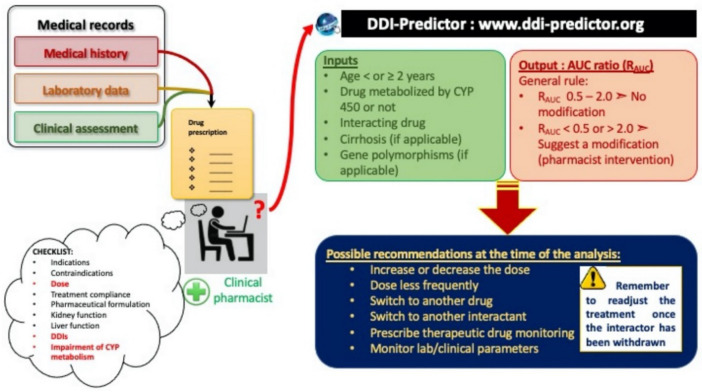
The DDI screening process.

The DDI screening process is shown schematically in Figure 2. Three different tools were used. If a DDI was suspected, the clinical pharmacist consulted the regularly updated ANSM thesaurus. This tool was integrated into our prescription analysis software. Other tools can also be used, including the GUH table and DDI-Predictor. Depending on the patient’s clinical background, a PI is sent to the attending physician. In the present study, 11 clinical pharmacists and 9 pharmacy residents analyzed prescriptions. All had received training in the use of these tools.

### 4.2. Description of the Tools Used

The thesaurus published by the ANSM is the French national reference for detecting DDIs [15]. It classes DDIs according to the iatrogenic risk encountered by patients if the drug combination is prescribed. There are four risk levels: a contraindicated drug combination, an at-risk drug combination, a drug combination to be administered with caution, and an interaction to be taken into consideration. As this tool has been integrated into our computerized physician order entry system, it can be used directly for medication analyses by our clinical pharmacists. If one of the four grades of DDI is detected, other tools can be used to deepen the analysis. Furthermore, if the DDI is not detected by the tool but is strongly suspected by the pharmacist, the GUH table and DDI-Predictor can also be applied.

The GUH table lists (i) the biotransformation pathways of drugs involving the main CYPs and P-gp, (ii) the main inhibitors of these enzymes, and (iii) the main inducers. The table is based on a review of the literature data from in vivo and in vitro studies. The magnitude of the biotransformation (major or minor) and the strength of the induction or inhibition is given for each drug. [16] This tool is notably of value for completing the data given in the SPC.

The DDI-Predictor application was created by the Genophar working group of the University-Hospital from Lyon, France. This group is composed of clinical pharmacists, pharmacologists, modelers, a biologist and a computer scientist. The website (www.ddi-predictor.org, accessed on 18 January 2021) was launched in 2013. The working group regularly updates the data and external evaluations. DDI-Predictor targets the main CYP isoforms implicated in the drug metabolism (3A4, 2D6, 2C9, 2C19 and 1A2). This application was used when a medication adjustment could be envisaged in the case of DDIs identified by the ANSM thesaurus when precisions were not available or sufficient on the SPC or when the DDIs was not detected by the ANSM thesaurus but the GUH tool contributed to identify such DDI.

DDI-Predictor comprises five modules. The first three are (a) interaction between two drugs, (b) the drug exposure level in cases of cirrhosis, and (c) the drug exposure level in patients with polymorphisms in the genes coding for CYP2D6, 2C9 and 2C19. The fourth and fifth modules result from combining (a) + (b) and (a) + (c), respectively. Moreover, DDI-Predictor can even? also be used for prescriptions in young children (under the age of 2, i.e., when the enzyme system is maturing. The lists of substrates, inhibitors and inducers are predefined.

DDI-Predictor’s algorithm is based on steady-state equations in a physiologically based pharmacokinetic model [30]. The model has been externally validation ([17] and on the tool’s web site). The parameters for substrates and interactors were estimated exclusively from clinical studies: no in vitro data were used [30].

The analyses were performed as follows. On the DDI-Predictor website, the operator entered the patient’s age (< or ≥2 years), the name and dose of the substrate (i.e., the “victim drug”) and the name and dose of the inhibitor or inducer (the “interactor”). The DDI-Predictor algorithm then computed the R_AUC_, as follows:

R_AUC_ = AUC for the substrate administered with the interactor/AUC for the substrate administered alone (1)

The R_AUC_’s 95% tolerance interval was also given [31].

Depending on the magnitude of the R_AUC_, the pharmacist should suggest one or more the following medication adjustments: a dose increase, a change in the substrate, a dose decrease, less frequent dosing, a change in the interactor; therapeutic drug monitoring; adverse drug reaction monitoring, or withdrawal of the interactor (Figure 2). The following equation was used to calculate the dose adjustment:

Adjusted dose = Current dose/R_AUC_ (2)

The same equation was used for prodrugs because the R_AUC_ corresponds to the active metabolite moiety in such cases.

### 4.3. Data Collection

Data were continuously acquired during routine computer-assisted drug prescription for inpatients in our institution. After being anonymized, the data were recorded in a standardized Excel^®^ spreadsheet (Microsoft Corporation, Redmond, WA, USA). The data analyzed in the present study were generated over 24 consecutive months. The department’s pharmacists were trained by two expert pharmacists (FM and NS), who had initially been trained by the DDI-Predictor’s developers. Each pharmacist was asked to present a clinical case involving DDI-Predictor, in order to check that the application was being used correctly. Moreover, quality assurance documents (for correct use of DDI-Predictor and help in interpretating the results) were created at the start of the study.

After using DDI-Predictor, the pharmacist filled out a care report form. In addition to the date, ward, drug, dosage regimen, and the interactor, the pharmacist noted whether a PI had been issued (yes/no) and, if so, gave details of the intervention: a change of drug (yes/no, and the name of the replacement, if applicable), a change of interactor (if applicable), a dose modification (a dose increase, a dose decrease, or a change in dosing frequency), therapeutic drug monitoring, and patient monitoring for adverse drug reactions. The physician’s acceptance (yes/no) of the PI was then recorded. Each PI had to be justified by the pharmacist, according to a predetermined list: addition of pharmacological effects, maximum dose reached or exceeded, limited clinical impact, lack of efficiency, a fragile patient, or a risk of overdosing. Lastly, the absence of a PI also had to be justified-notably when several interacting drugs or any factors possibly causing of a pharmacokinetic alteration (kidney failure, obesity, etc.) were present.

### 4.4. Data Coding

Each tool categorizes the strength or risk level of each detected DDI.

According to the ANSM thesaurus, the strength/risk level of a DDI is categorized as 1: a contraindicated drug combination; 2: an at-risk drug combination; 3: a drug combination to be administered with caution; or 4: an interaction to be taken into consideration

The GUH tables classify DDIs into 5 groups, depending on the combination of weak or strong interactors (i or I, respectively) with weak or strong metabolized drugs (m or M, respectively): I/M, I/m, M/I, i/m, or no interaction.

Lastly, the mean value of R_AUC_ provided by DDI-Predictor was categorized as R_AUC_ ≤ 0.5, R_AUC_ 0.5–2 or R_AUC_ > 2. R_AUC_ ≤ 0.5 and R_AUC_ > 2 correspond to at-risk DDIs.

For each tool, interactions that did not fit into any of the defined categories were noted as “not recorded”.

### 4.5. Data Analysis

Firstly, the tools’ respective DDI detection rates (the number of DDIs reported by each tool, divided by the total number of DDIs recorded during the study, in %) were compared in a chi-squared test. Secondly, the data produced by DDI-Predictor were described, and the PIR (as a percentage of the analyzed events) and the PAR (as a percentage of the PIs issued) were computed. Each type of PI was characterized with the same parameters. The cases were analyzed according to whether the interacting agent was and enzyme inducer or an enzyme inhibitor. The PIR and PAR were also analyzed according to the type of interacting agent and were compared using a chi-squared test. The threshold for statistical significance was set to *p* < 0.05.

Lastly, misuse of DDI-Predictor was identified when the pharmacist either (i) highlighted a DDI although the criteria defined in the application were not met, or (ii) misinterpreted the R_AUC_. In order quantify the misuse rate over the whole studied period, each case was reviewed by a pharmacist with expertise in DDI-Predictor (FM).

## 5. Conclusions

The present study is the first to have described the implementation and use of DDI-Predictor in routine clinical practice. This web-application is very easy to use, although pharmacists must be trained to interpret the results correctly-notably with regard to the patient’s clinical status at the time of the analysis. DDI-Predictor flags up the potential occurrence of adverse drug reactions and gives the pharmacist more information for resolving a drug treatment problem. This decision support provided by DDI-Predictor usefully complements other tools. Further studies are necessary to refine our practice and to identify factors that might improve PIs and increase the PAR.

## Figures and Tables

**Table 1:** Comparison of the three tools’ respective abilities to detect DDIs. Numbers not in italics refer to DDIs involving enzyme inducers, whereas numbers in italics refer to DDIs involving enzyme inhibitors. According to the ANSM thesaurus, the strength/risk level of a DDI is categorized as 1: a contraindicated drug combination; 2: an at-risk drug combination; 3: a drug combination to be administered with caution; or 4: an interaction to be taken into consideration. In the GUH table, weak and strong interactors are referred to as “i” or “I”, respectively, and weakly and strongly metabolized drugs are referred to as “m” or “M”, respectively. The strength of the DDI is then categorized as I/M (1), I/m (2), M/i (3), i/m (4), ND (not detected) or NR (not recorded). The R_AUC_ is quoted as the mean ± standard deviation.

ANSM		GUH						DDI-P				
-	N	1	2	3	4	ND	NR	N	R_AUC_ ≤ 0.5	R_AUC_ 0.5–2	R_AUC_ > 2	NR
1	0	0	0	0	0	0	0	0	-	-	-	-
	*4*	*2*	*0*	*0*	*0*	*0*	*2*	*4*	*-*	*-*	*3.44* ± *1.15*	*0*
2	12	8	3	0	0	0	1	10	0.22 ± 0.06	1	*-*	2
	*10*	*9*	*0*	*0*	*0*	*0*	*1*	*9*		*1.74*	*6.10* ± *5.47*	*1*
3	18	9	1	4	0	2	2	15	0.31 ± 0.06	0.69 ± 0.12	*-*	3
	*26*	*18*	*2*	*2*	*0*	*0*	*4*	*21*	*-*	1.71 ± 0.38	*5.03* ± *1.97*	*5*
4	2	0	0	0	0	2	0	1	0.5	-	*-*	1
	*20*	*5*	*8*	*4*	*2*	*0*	*1*	*19*	*-*	1.47 ± 0.1	*2.56* ± *0.84*	*1*
ND	35	15	2	0	1	5	12	29	0.29 ± 0.04	0.765 ± 0.24	*-*	6
	*100*	*44*	*22*	*12*	*3*	*10*	*9*	*85*	*-*	*1.46* ± *0.07*	*4.17* ± *1.14*	*15*
NR	0	0	0	0	0	0	0	0	-	-	*-*	0
	*3*	*0*	*0*	*0*	*0*	*0*	*3*	*3*	*-*	*1.19* ± *1.97*	*6.13*	*0*

**Table 2:** Description of the type of PI and the PAR. ^†^ *p* < 0.0001; ^‡^ *p* < 0.729.

	R_AUC_ ≤ 0.5		R_AUC_ 0.5–2		R_AUC_ > 2	
Median	0.28		1.51		3.05	
	N	%	N	%	N	%
**PIs**	36/40	**90.0 ^†^**	46/104	**44.2 ^†^**	39/52	**75.0 ^†^**
*Dose increase*	8/9	**88.9**	1/1	**100**	0/0	**-**
*Change in the substrate*	13/15	**86.7**	0/0	**-**	7/7	**100**
*Dose decrease*	0/0	**-**	4/6	**66.7**	11/12	**91.7**
*Less frequent dosing*	1/1	**100**	0/0	**-**	0/0	**-**
*Change in the interactor*	0/4		2/2	**100**	3/3	**100**
*Therapeutic drug monitoring*	4/4	**100**	8/9	**88.9**	2/4	**50**
*Adverse drug reaction monitoring*	5/5	**100**	24/28	**86**	9/13	**69**
*Withdrawal of the interactor*	1/2	**50**	-	-	-	-
**PAR**	32/36	**88.8 ^‡^**	39/46	**84.8 ^‡^**	32/39	**82.0 ^‡^**

**Table 3:** Comparison of PIs and PARs for DDI-Predictor, the ANSM thesaurus and the GUH tables. ^a^ *p* < 0.05; ^b^ *p* < 0.001; ^c^ *p* < 0.001.

	R_AUC_ ≤ 0.5 (N = 36)		R_AUC_ 0.5–2 (N = 46)		R_AUC_ > 2 (N = 39)		Total
Detected	PIs	PAR	PIs	PAR	PIs	PAR	-
ANSM	13 ^a^ (36.1%)	13/13	17 ^b^ (37.0%)	15/17	20 ^c^ (51.3%)	13/20	50 (41.3%)
GUH	25 ^a^ (69.4%)	21/25	37 ^b^ (80.4%)	31/37	36 ^c^ (92.3%)	30/36	98 (81.0%)
Not detected	-	-	-	-	-	-	-
ANSM	23 (63.9%)	19/23	29 (63.0%)	24/29	19 (48.7%)	17/19	71 (58.7%)
GUH	11 (30.6%)	11/11	9 (19.6%)	8/9	3 (7.7%)	2/3	23 (19.0%)

## Data Availability

The data presented in this study are available on request from the corresponding author. The data are not publicly available due to their potential reuse for further analyses and publications.
